# Sub-genomic flaviviral RNA elements increase the stability and abundance of recombinant AAV vector transcripts

**DOI:** 10.1128/jvi.00095-24

**Published:** 2024-07-31

**Authors:** Rita M. Meganck, Roza Ogurlu, Jiacheng Liu, Sven Moller-Tank, Victor Tse, Leo O. Blondel, Alan Rosales, Aaron C. Hall, Heather A. Vincent, Nathaniel J. Moorman, William F. Marzluff, Aravind Asokan

**Affiliations:** 1 Department of Surgery, Duke University School of Medicine, Durham, North Carolina, USA; 2 Gene Therapy Center, University of North Carolina at Chapel Hill, Chapel Hill, North Carolina, USA; 3 Department of Molecular Microbiology, Washington University in St. Louis School of Medicine, St. Louis, Missouri, USA; 4 Department of Biomedical Engineering, Duke University, Durham, North Carolina, USA; 5 Cornell University, Ithaca, New York, USA; 6 Regeneron Pharmaceutical, Inc., Tarrytown, New York, USA; 7 Saint Louis University School of Medicine, St. Louis, Missouri, USA; 8 Department of Cell Biology & Physiology, University of North Carolina at Chapel Hill, Chapel Hill, North Carolina, USA; 9 TorqueBio, Inc., Durham, North Carolina, USA; 10 Department of Microbiology & Immunology, University of North Carolina at Chapel Hill, Chapel Hill, North Carolina, USA; 11 Department of Biochemistry & Biophysics, University of North Carolina at Chapel Hill, Chapel Hill, North Carolina, USA; 12 Department of Molecular Genetics & Microbiology, Duke University School of Medicine, Durham, North Carolina, USA; Cornell University Baker Institute for Animal Health, Ithaca, New York, USA

**Keywords:** flavivirus, AAV vectors, expression, mRNA stability, translation, gene therapy

## Abstract

Many viruses have evolved structured RNA elements that can influence transcript abundance and translational efficiency, and help evade host immune factors by hijacking cellular machinery during replication. Here, we evaluated the functional impact of sub-genomic flaviviral RNAs (sfRNAs) known to stall exoribonuclease activity, by incorporating these elements into recombinant adeno-associated viral (AAV) genome cassettes. Specifically, sfRNAs from Dengue, Zika, Japanese Encephalitis, Yellow Fever, Murray Valley Encephalitis, and West Nile viruses increased transcript stability and transgene expression compared to a conventional woodchuck hepatitis virus element (WPRE). Further dissection of engineered transcripts revealed that sfRNA elements (i) require incorporation in *cis* within the 3′ untranslated region (UTR) of AAV genomes, (ii) require minimal dumbbell structures to exert the observed effects, and (iii) can stabilize AAV transcripts independent of 5′−3′ exoribonuclease 1 (XRN1)-mediated decay. Additionally, preliminary *in vivo* assessment of AAV vectors bearing sfRNA elements in mice achieved increased transcript abundance and expression in cardiac tissue. Leveraging the functional versatility of engineered viral RNA elements may help improve the potency of AAV vector-based gene therapies.

**IMPORTANCE:**

Viral RNA elements can hijack host cell machinery to control stability of transcripts and consequently, infection. Studies that help better understand such viral elements can provide insights into antiviral strategies and also potentially leverage these features for therapeutic applications. In this study, by incorporating structured flaviviral RNA elements into recombinant adeno-associated viral (AAV) vector genomes, we show improved AAV transcript stability and transgene expression can be achieved, with implications for gene transfer.

## INTRODUCTION

Adeno-associated virus (AAV) vectors are promising vectors for gene therapy-based treatment of monogenic and complex genetic diseases (1). AAV is a non-pathogenic dependoparvovirus with a single-stranded DNA genome with *Rep* and *Cap genes* flanked by two inverted terminal repeat (ITR) regions (2). The ITRs are the only genomic elements essential for packaging any transgene of interest in a recombinant AAV vector (3). Both natural and synthetic AAV capsids have been used to deliver genetic cargo to target tissues in preclinical studies and clinical trials (1, 4). Notably, recent FDA approvals of Hemgenix, Zolgensma, Luxturna, and Roctavian for hemophilia B, spinal muscular atrophy (SMA), inherited retinal disease, and severe hemophilia A, respectively, underscore the translational potential of AAV gene therapy vectors (4–8).

Different components of the recombinant AAV genome such as promoter elements, introns, short hairpins, riboswitches, and other regulatory elements such as microRNA binding sites have been investigated by several groups (9–15). However, the vast functional diversity of post-transcriptional regulatory RNA elements remains largely under-explored in AAV vector design. For instance, the woodchuck hepatitis post-transcriptional regulatory element (WPRE) was incorporated into recombinant viral vector genomes to improve yield and transgene expression (16–18). Although also commonly incorporated in AAV vector genomes (9, 19), a consistent and efficient role for WPRE in improving transcript stability or nuclear export to improve gene expression *in vivo* has not been demonstrated. Additionally, post-transcriptional regulatory mechanisms employed by other viruses present potential new opportunities in the context of AAV vectors.

A particularly intriguing example in this regard is the *Flavivirus* genus comprised of small enveloped viruses with a positive-stranded RNA genome that contains a complex RNA secondary structure at their 3′ end instead of a polyA tail (20, 21). Specifically, in flavivirus-infected cells, the genomic RNA (gRNA) undergoes 5′ to 3′ exonucleolytic degradation by 5′−3′ exoribonuclease 1 (XRN1), releasing ~300–500 nucleotides of the 3′ end of gRNA to produce a sub-genomic flaviviral RNA (sfRNA). During infection, sfRNAs accumulate to high levels in the cell and contribute to flavivirus infection through multiple mechanisms (20, 22); specific sfRNA structures stall not only XRN1 but also other 5′−3′ exonucleases and alter mRNA stability dynamics (23–25), sfRNA binds to a variety of host proteins that play roles in viral replication and translation (26–29), and there is growing evidence that the sfRNAs are involved in blocking host innate immune response (30–32). Thus, we decided to explore the potential functional impact of sfRNA elements in the context of rAAV transcript biology.

Specifically, we show that sfRNA elements from different flaviviruses enhance rAAV-mediated transgene expression, with the sfRNA element from Dengue virus serotype 2 (DENV2) exerting the greatest effect. This function was dependent on the sfRNA element being present in the 3′ untranslated region (UTR) of the AAV transcript, and we further show that the two dumbbell RNA structures within the DENV2 sfRNA are necessary and sufficient to increase AAV gene expression. Interestingly, unlike the flaviviral context, this effect was XRN1 independent, resulting in increased AAV transcript stability that promoted increased transgene expression. Together, these results highlight the utility of sfRNA elements in enhancing rAAV transcript stability and support further exploration of other viral post-transcriptional regulatory elements that might augment AAV-mediated gene transfer.

## RESULTS

### Flaviviral sfRNA elements enhance AAV transgene expression efficiency *in vitro*

**Fig 1:**
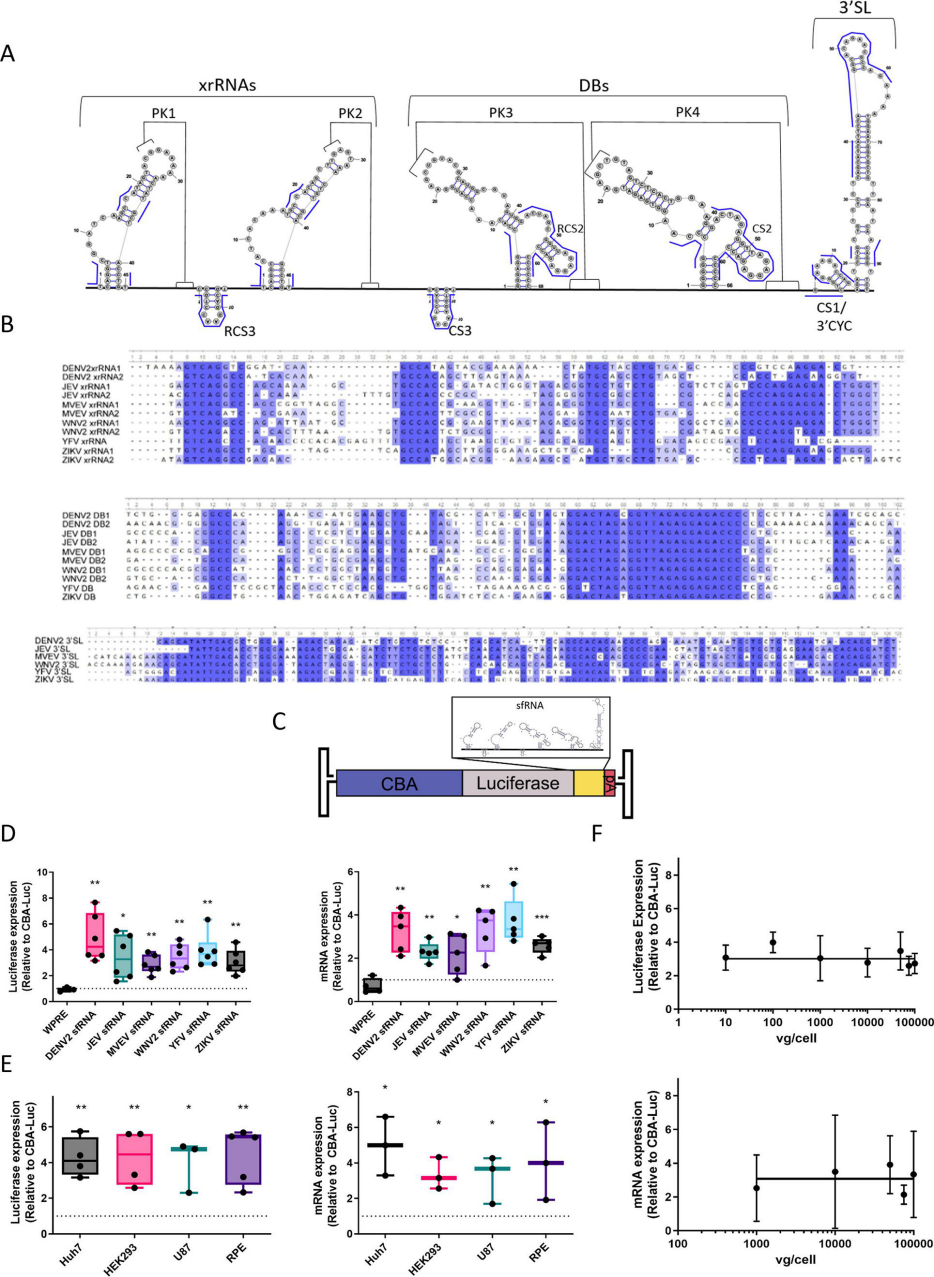
Subgenomic flaviviral RNAs increase transgene expression. (**A**) Secondary structure of the DENV2 sfRNA. Conserved sequences are overlaid in blue, and various structural elements are noted. CS1/3’CYC: conserved sequence 1, the 3′ cyclization sequence. CS2: conserved sequence 2. CS3: conserved sequence 3. (**B**) Alignments of xrRNA, DB, and 3′SL structures from DENV2, JEV, MVEV, WNV2, YFV, and ZIKV. (**C**) Diagram of the experimental constructs. A luciferase transgene with SV40 polyA signal and driven by the CBA promoter was placed between AAV2 ITRs. SfRNA (or WPRE) sequences were placed as the 3′UTR of the luciferase mRNA. (**D**) Huh7 cells were transduced with AAV2 packaging the above constructs at 10,000 vg/cell and harvested at 3 d post-transduction. Lysates were measured for relative luciferase expression (RLUs). RNA from replicate samples was used to perform RT-qPCR for luciferase mRNAs. (**E**) The indicated cell lines were transduced with 10,000 vg/cell of AAV2 vectors packaging a DENV2 sfRNA construct and harvested at 3 d post-transduction. Lysates were measured for relative luciferase expression (RLUs). RNA from replicate samples was used to perform RT-qPCR for luciferase mRNAs. (**F**) Huh7 cells were transduced with AAV2 encoding normal CBA-Luc or with a 3′DENV2 sfRNA at indicated MOIs. Cells were harvested 3 d post-transduction. Lysates were measured for relative luciferase expression (RLUs). RNA from replicate samples was used to perform RT-qPCR for luciferase mRNAs. For all graphs, samples are plotted relative to a CBA-Luc transgene with no additional 3′UTR. Where indicated, * = *P* < 0.05; ** = *P* < 0.005; *** = *P* < 0.0005.

We selected sfRNAs from six flaviviruses: Dengue virus serotype 2 (DENV2), Japanese Encephalitis virus (JEV), Murray Valley Encephalitis virus (MVEV), West Nile virus serotype 2 (WNV2), Yellow Fever virus (YFV), and Zika virus (ZIKV). Although the sfRNA structures are conserved among the different viruses, there is limited sequence conservation (23, 33). When aligning the sequences of the structural elements and subsequent mapping onto a secondary structure model of the DENV2 sfRNA, several conserved “patches” are found in the stems of the XRN1-resistant (xrRNA) hairpins and in loop portions of the dumbbell structures (DBs) (Fig. 1A). Notably, the bases that form the pseudoknots in both the xrRNAs and DBs have little to no conservation at the sequence level (Fig. 1B), although their tertiary structure is known to be important for sfRNA function (21).

We inserted each of the above sfRNA sequences into the 3′UTR of a polyadenylated luciferase reporter encoded in an AAV ITR-flanked vector cassette (Table 1; Fig. 1C). The sfRNAs were generally placed 40–60 nts after the stop codon and ended 40–45 nts before the polyadenylation signal depending on each construct. In this system, the reporter is transcribed from the chicken β-actin (CBA) promoter. We compared these constructs to a standard luciferase reporter with an SV40 polyadenylation signal, as well as a reporter with a woodchuck hepatitis post-transcriptional regulatory element (WPRE) inserted at the same location as the sfRNAs. Addition of the WPRE in the 3′UTR in the context of the CBA promoter-driven cassettes had no effect on expression. It is important to note that transgene expression is not expected to be significantly increased by including WPRE, *in vitro* and *in vivo*, when driven from strong promoters with introns, e.g., EF1α or CAG/CBA (9). However, insertion of each of the six sfRNAs increased both luciferase and mRNA expression by three- to fivefold (Fig. 1D). As the effect was slightly greater with DENV2 sfRNA, we chose to focus on this RNA element for subsequent experiments. First, we tested this construct in four human cell lines: Huh7 (hepatocarcinoma), HEK293 (embryonic kidney), U87 (glioblastoma), and RPE (retinal pigment epithelium). In every cell line, we observed a comparable increase in expression (Fig. 1E), corroborating that the enhanced effect observed with the DENV2 sfRNA is likely supported by broadly conserved cellular machinery. Notably, the DENV2 sfRNA construct delivered by AAV2 vectors at different multiplicities of infection (MOIs) outperformed the conventional vector without a 3′UTR by three- to fivefold (luciferase expression and mRNA transcript levels) regardless of the MOI (Fig. 1F).

**TABLE 1:** Summary of different genome configurations included in the current study^*a*^

Construct	Serotype	3′UTR length (nt)	rAAV genome length (nt)	Titer (vg/µL)
CBA-Luc-SV40 polyA	2	40	3,825	5.69E8
CBA-Luc-WPRE-SV40 polyA	2	642	4,427	3.64E8
CBA-Luc-DENV2 sfRNA-SV40 polyA	2	510	4,384	4.23E8
CBA-Luc-JEV sfRNA-SV40 polyA	2	621	4,406	5.24E8
CBA-Luc-MVEV sfRNA-SV40 polyA	2	622	4,407	6.17E8
CBA-Luc-WNV2 sfRNA-SV40 polyA	2	627	4,412	4.61E8
CBA-Luc-YFV sfRNA-SV40 polyA	2	431	4,216	9.93E8
CBA-Luc-ZIKV sfRNA-SV40 polyA	2	478	4,263	9.91E8
CBA-DENV2 sfRNA-Luc-SV40 polyA	2	40	4,303	1.09E9
U6-DENV2 sfRNA-CBA-Luc-SV40 polyA	2	40	4,593	8.33E8
CBA-Luc-DENV2ΔxrRNA-SV40 polyA	2	333	4,088	3.62E8
CBA-Luc-DENV2ΔDB-SV40 polyA	2	351	4,136	5.46E8
CBA-Luc-DENV2Δ3’SL-SV40 polyA	2	385	4,140	3.95E8
CBA-Luc-DENV2 DB-SV40 polyA	2	266	4,051	3.51E8
CBA-Luc-DENV2 DB1-SV40 polyA	2	181	3,966	5.06E8
CBA-Luc-DENV2 DB1 mut-SV40 polyA	2	181	3,966	6.03E8
CBA-Luc-DENV2 DB1 Cmut-SV40 polyA	2	181	3,966	8.83E8
CBA-Luc-RiboJ	2	80	3,655	5.49E8
CBA-Luc-DENV2 sfRNA-RiboJ	2	537	4,112	6.24E8
CBA-Luc-WPRE-SV40 polyA	9	617	4,483	5.24E9
CBA-Luc-DENV2 sfRNA-SV40 polyA	9	485	4,351	3.57E9
CBA-Luc-DENV2 DB-SV40 polyA	9	241	4,107	5.74E9

^*a*^ Recombinant AAV vector serotype and titers are included for all preparations.

### Effect of sfRNA on AAV transgene expression is positionally dependent

**Fig 2:**
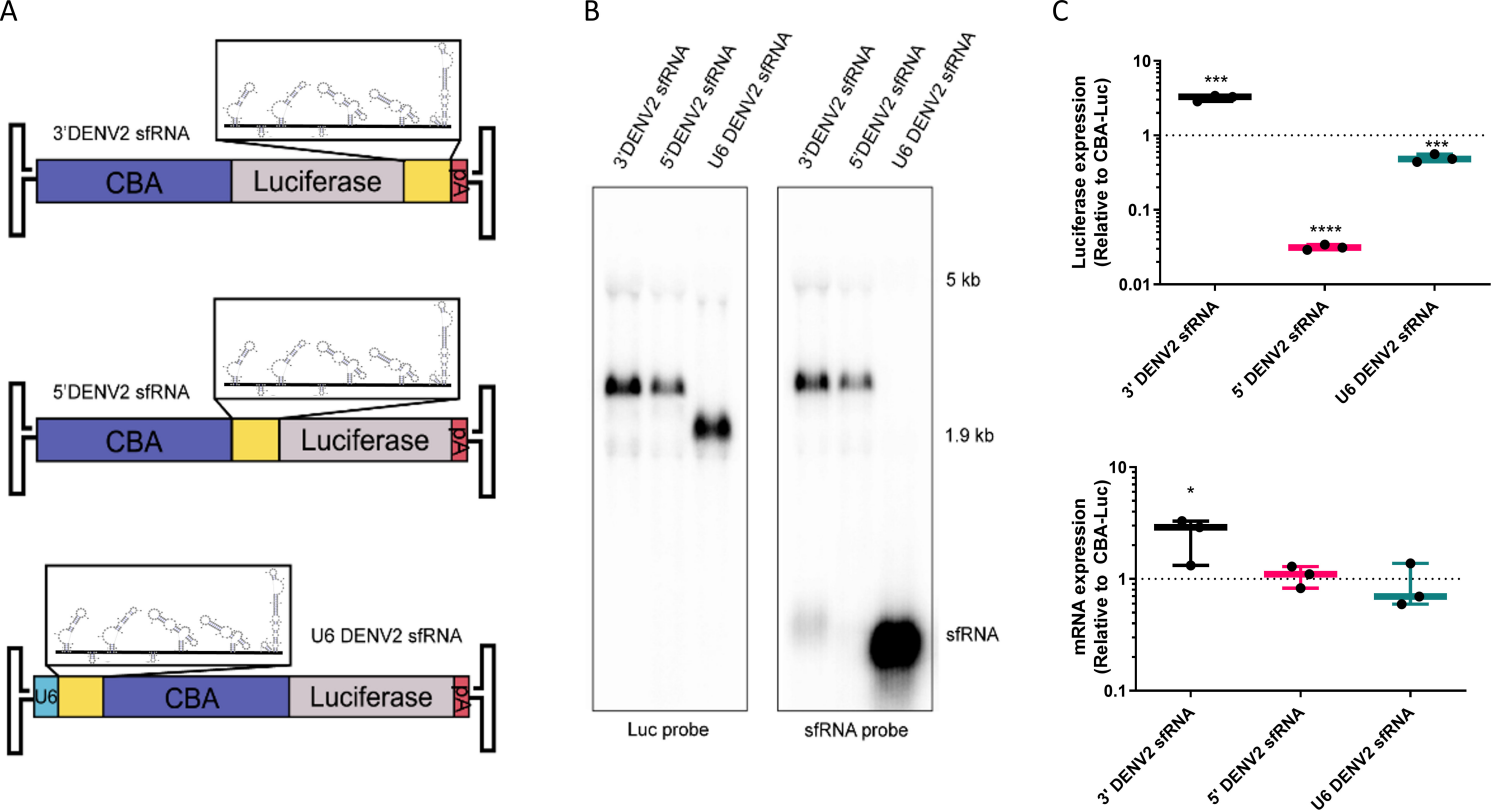
DENV2 sfRNA effect on transgene expression is positionally dependent. (**A**) The DENV2 sfRNA was placed in either the 3′UTR (top), 5′UTR (middle), or as a separate, U6-driven RNA packaged in the same AAV genome (bottom). (**B**) The indicated constructs were transfected into HEK293 cells and harvested 3 d post-transfection. RNA was used to run a Northern blot and probed for either luciferase or sfRNA sequences. (**C**) Huh7 cells were transduced with the indicated constructs at 10,000 vg/cell of AAV2 and harvested 3 d post-transduction. Top: lysates were measured for relative luciferase expression (RLUs). Bottom: RNA from replicate samples was used to perform RT-qPCR for luciferase mRNAs. Data are shown relative to a CBA-Luc transgene with no additional 3′UTR. Where indicated, * = *P* < 0.05; ** = *P* < 0.005; *** = *P* < 0.0005; **** = *P* < 0.00005.

We further investigated whether the increase in expression was a *cis* or *trans* effect. We originally placed the sfRNA in the 3′UTR of our transgene, consistent with its location within flavivirus genomes. We then generated additional constructs placing the sfRNA in the 5′UTR of the luciferase gene, or under the control of a separate U6 promoter in the same AAV genome as the luciferase gene (Fig. 2A). DENV2 sfRNA expression from these reporters was then determined by Northern blot, probing for either the luciferase sequence or the DENV2 sfRNA. The 3′ sfRNA construct produced primarily full-length mRNA, with a small amount of sfRNA observed, whereas the 5′ sfRNA construct produced only a full-length mRNA product. We also confirmed that the U6-sfRNA construct did indeed produce large amounts of sfRNA (Fig. 2B). We next tested the gene expression efficiency of these AAV reporters compared to the standard polyadenylated luciferase reporter. Similar to what we previously observed, inclusion of the sfRNA in the 3′UTR increased luciferase mRNA and gene expression. When the sfRNA was placed in the 5′UTR, RNA levels were similar to the standard luciferase reporter; however, luciferase expression levels dropped to near background levels, possibly due to translational blockade by the highly structured sfRNA. With *trans* expression of U6-driven sfRNAs, luciferase RNA levels were similar to the original construct, and gene expression was slightly reduced (Fig. 2C). These data reveal that the increase in expression arises from the *cis* effect of sfRNA when placed specifically in the 3′UTR.

### sfRNA dumbbell elements (DBs) are essential for increased AAV transgene expression

**Fig 3:**
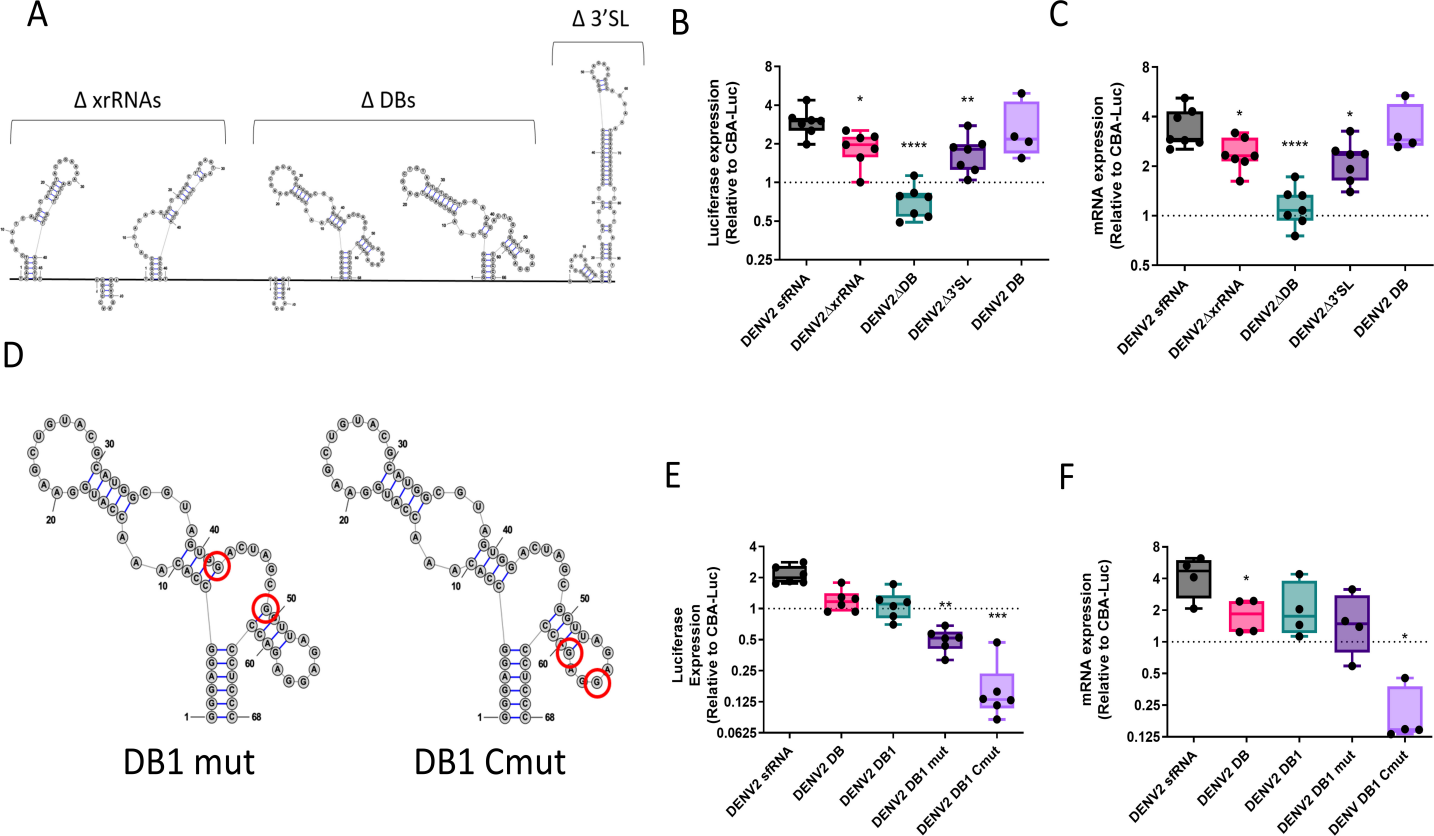
The DENV2 DB elements are sufficient and necessary to increase transgene expression. (**A**) Diagram of the DENV2 sfRNA, with the indicated deletions of RNA structural elements. (**B**) Huh7 cells were transduced with AAV2 packaging sfRNA constructs with the indicated deletions at 10,000 vg/cell and harvested at 3 d post-transduction. Lysates were measured for relative luciferase expression (RLUs). (**C**) RNA from replicate samples was used to perform RT-qPCR for luciferase mRNAs. (**D**) Mutations in the first DB of DENV2 sfRNA were created at the indicated locations. (**E**) Huh7 cells were transduced with the mutated constructs at 10,000 vg/cell of AAV2 vector and collected at 3 d after transduction. Lysates were measured for relative luciferase expression (RLUs). (**F**) RNA from cells was used to perform RT-qPCR for luciferase mRNAs. Data are shown relative to a CBA-Luc transgene with no additional 3′UTR for all plots. Where indicated, * = *P* < 0.05; ** = *P* < 0.005; *** = *P* < 0.0005; **** = *P* < 0.00005.

In mosquito-borne Flaviviruses, there are three major structural RNA elements present in the sfRNA: XRN1-resistant RNAs (xrRNAs) at the 5′ end, dumbbell RNAs (DBs) in the center, and a terminal 3′ stem loop (3′SL) (21–23). Correspondingly, we designed constructs that lacked the xrRNA elements, DB elements, or the 3′SL (ΔxrRNAs, ΔDBs, and Δ3′SL, respectively), while attempting to preserve the structures of the remaining RNA elements (Fig. 3A). We then tested the effect of these deletions on gene expression efficiency. Deletion of either the xrRNAs or 3′SL had only a mild effect, whereas deletion of the DBs reduced expression at both the protein and RNA levels to a level comparable to luciferase mRNA lacking an insertion in the 3′UTR (Fig. 3B and C). This result indicated that the DB elements are necessary for increasing RNA levels. We then tested a construct containing only the DENV2 DB elements in the 3′UTR and found that these enhanced protein and mRNA expression to levels higher than control and only slightly lower than that of a construct containing the full-length sfRNA (Fig. 3B and C). Thus, the DENV2 DBs are necessary and sufficient for increasing AAV gene expression.

We further investigated the role of the sfRNA DBs by introducing mutations in the cyclization motif (G → A; DB1 mut) and in one of the dumbbell arms (G → C; DB1 Cmut) (Fig. 3D). We found that a single DB element was sufficient to increase both protein and mRNA to levels higher than control and slightly reduced compared to constructs containing both DBs and the full-length sfRNA (Fig. 3E and F). Interestingly, the G → A mutations (DB1 mut) had no effect on RNA levels, but resulted in a significant decrease in luciferase expression, implying a potential impact on translation. In contrast, the second set of mutations (DB1 Cmut) decreased RNA levels to lower than those seen with no sequence inserted (Fig. 3E and F), essentially converting the DB element from a stabilizing element to a destabilizing element. Although the underlying mechanisms driving these changes remain to be determined, these studies define the critical role of the DB structure in transcript stability and translation.

### sfRNA-dependent increases in AAV transgene expression are XRN1 independent

**Fig 4:**
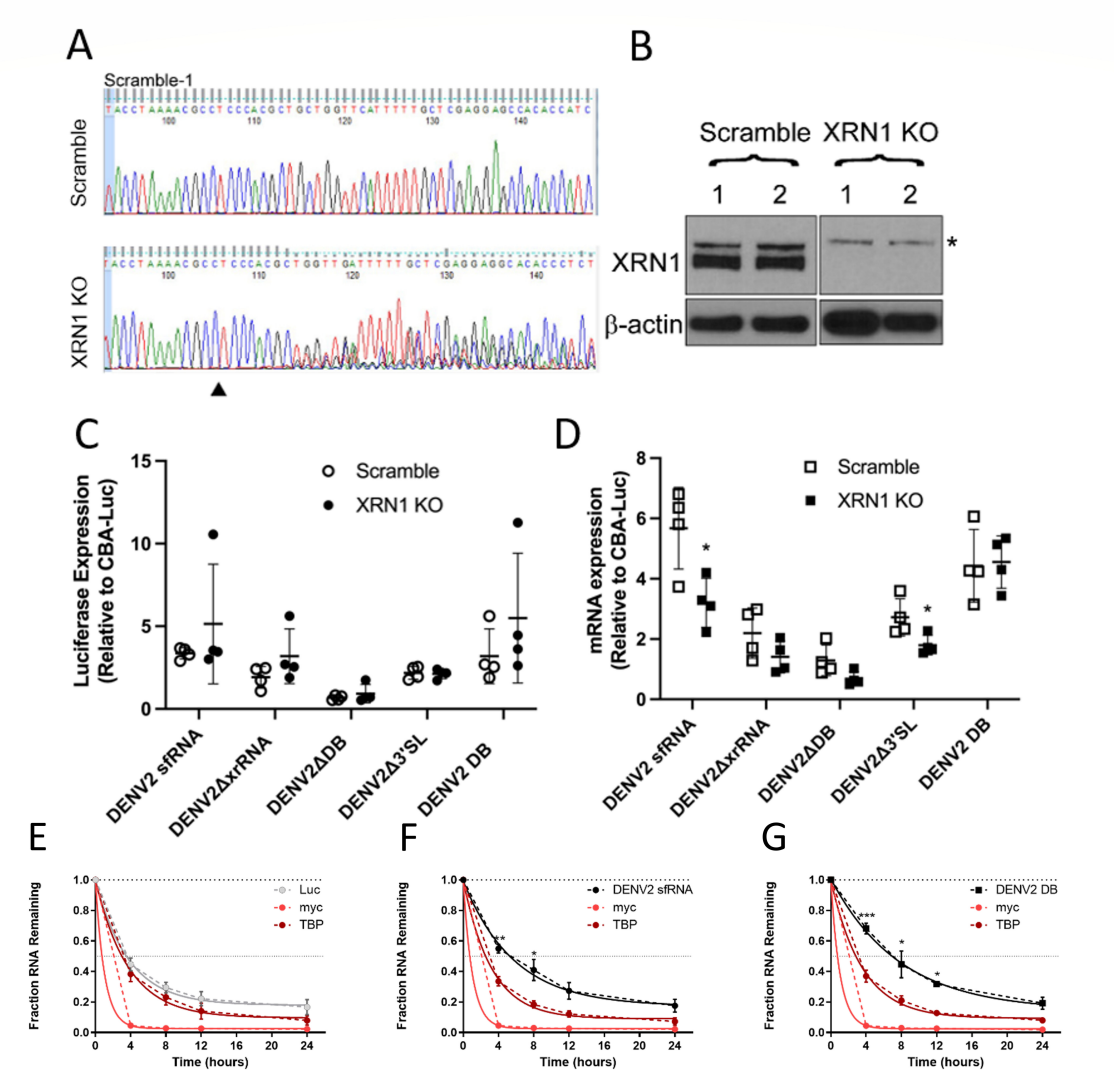
DENV2 sfRNA and DB element increase transcript stability in an XRN1-independent manner. (**A**) Huh7 cells were infected with lentivirus packaging Cas9 and either a Scramble gRNA or gRNA against the XRN1 gene. Following antibiotic selection, sequencing at the XRN1 locus confirmed Cas9 activity with the XRN1-1 gRNA. The arrow denotes the predicted Cas9 cleavage site. (**B**) Western blotting was performed on two independent Scramble and XRN1 KO clonal cell lines for XRN1 (top, * denotes nonspecific band) and β-actin (bottom). Scramble or KO cells were transduced with the indicated constructs at 10,000 vg/cell of AAV2 and harvested at 3 d post-transduction. (**C**) Lysates were measured for relative luciferase expression (RLUs). (**D**) RNA from replicate samples was used to perform RT-qPCR for luciferase mRNAs. Data are shown relative to a CBA-Luc transgene with no additional 3′UTR. Luciferase transgene with (**E**) no 3′UTR, (**F**) DENV2 3′UTR, and (**G**) DENV2 DB 3′UTR was packaged into AAV2. HEK293 cells were transduced with the constructs, and at 3 d post-transduction, actinomycin D was added to cells. RNA was collected at the indicated time points. RNA levels were measured by RT-qPCR, graphed relative to the level at 0 h and normalized to GAPDH mRNA levels. TBP and myc mRNAs were used as control. Solid lines indicate nonlinear regression fit of the data. Where indicated, * = *P* < 0.05; ** = *P* < 0.005; *** = *P* < 0.0005.

During Flaviviral infection, sfRNA accumulates to very high levels, inhibiting cellular XRN1 function, and exerts global effects on RNA stability (20–25). To determine if the increase in AAV transcript levels was due to sfRNA-dependent XRN1 inhibition, we assessed transgene expression in XRN1 knockout (KO) cells. Scramble and XRN1 KO Huh7 cell lines were generated using CRISPR-Cas9 (Fig. 4A and B). It is important to note that XRN1 KO cells eventually arrested after several passages; nevertheless, we were able to perform experiments with early passage cell lines. We then transfected the Scramble or XRN1 KO cells with a luciferase construct containing either a full-length DENV2 sfRNA or DENV2 sfRNA lacking key structural elements. There was no difference in luciferase expression from our constructs between Scramble and XRN1 KO cells (Fig. 4C). Although we did observe small, yet significant decreases in mRNA levels from the constructs containing the full-length sfRNA and the Δ3′SL sfRNA (Fig. 4D) in XRN1 KO cells, luciferase expression remained unchanged, suggesting that the overall effect of sfRNA on transgene expression is XRN1 independent.

### DENV2 sfRNA and DB elements enhance AAV transcript stability

Because we observed increased transcript abundance in the presence of sfRNA, we hypothesized the addition of sfRNA to the transcript 3′UTR increases RNA stability. To test this, we transfected cells with either a luciferase construct containing no 3′UTR, a construct containing the full-length DENV2 sfRNA in the 3′UTR, or only the two DENV2 DB structures in the 3′UTR. We then treated HEK293 cells with actinomycin D to inhibit mRNA synthesis and measured mRNA levels for up to 24 h. We found that presence of the DENV2 sfRNA or the DENV2 DBs was sufficient to significantly increase the luciferase mRNA half-life, confirming that the DB elements are necessary and sufficient to stabilize the mRNA (Fig. 4E through G). The half-lives of measured endogenous mRNAs, TATA-box binding protein (TBP) and myc, were unaffected by the presence of sfRNA-containing luciferase RNAs.

**Fig 5:**
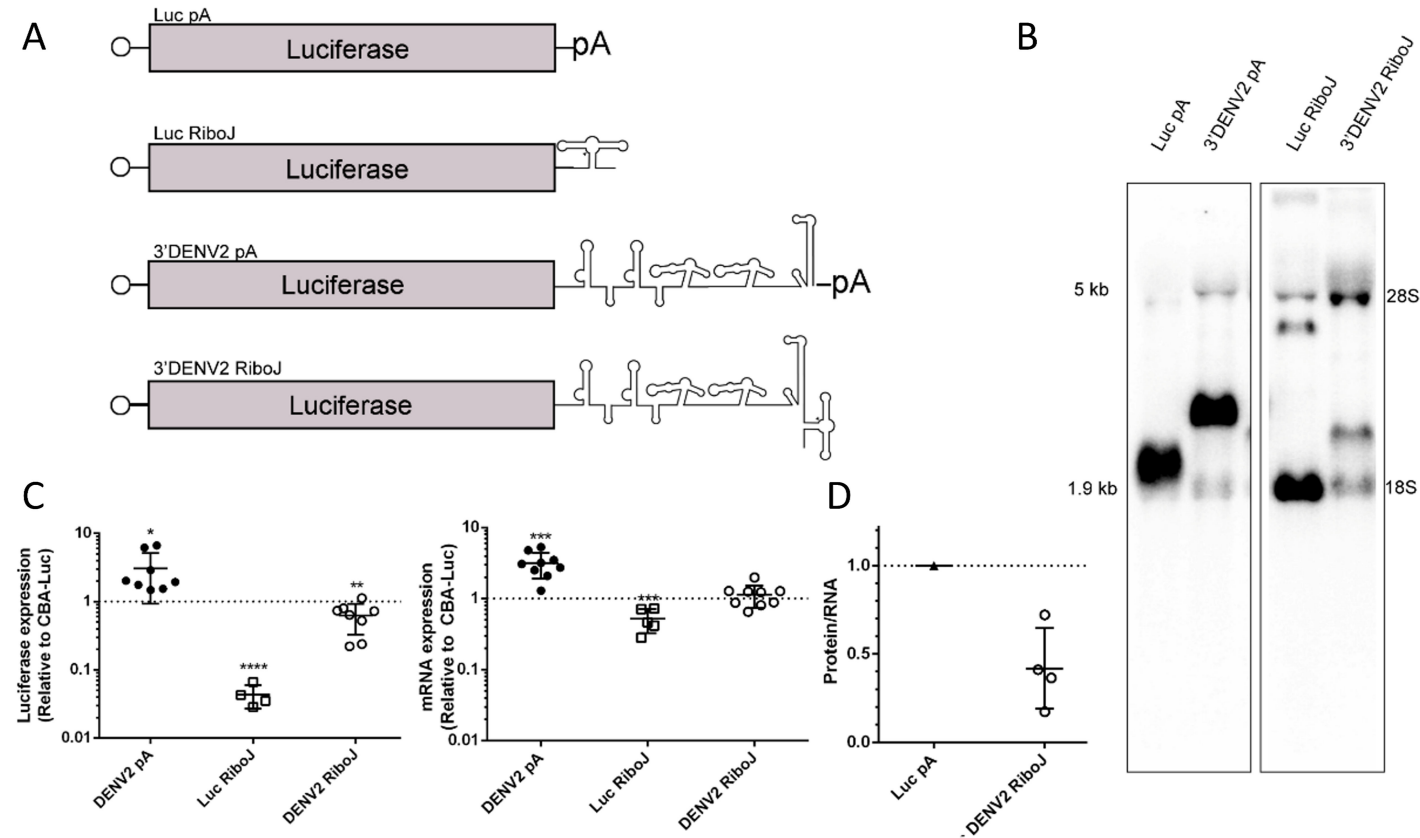
Presence of polyA tail in conjunction with sfRNA elements is necessary for increased transgene expression but not improved mRNA stability. (**A**) Diagram of luciferase mRNAs with either a polyA or RiboJ (hammerhead ribozyme) 3′ end, with and without the DENV2 sfRNA. (**B**) The indicated constructs were transfected into HEK293 cells and harvested 3 d post-transfection. RNA was used to run a Northern blot and probed for luciferase sequences. (**C**) Huh7 cells were transduced with the indicated constructs at 10,000 vg/cell and harvested at 3 d post-transduction. Left: lysates were measured for relative luciferase expression (RLUs). Right: RNA from replicate samples was used to perform RT-qPCR for luciferase mRNAs. Data are shown relative to a CBA-Luc transgene with no additional 3′UTR. (**D**) The ratio of protein and RNA expression was calculated for the DENV2 RiboJ construct, relative to CBA-Luc with a polyA tail. Student’s *t*-test was performed to test for statistical significance. Where indicated, * = *P* < 0.05; ** = *P* < 0.005; *** = *P* < 0.0005; **** = *P* < 0.00005.

To investigate whether the sfRNAs support stability and translation of AAV transcripts, we constructed Luciferase and 3′ DENV2 sfRNA constructs with hammerhead ribozyme RiboJ replacing the polyA signal (Fig. 5A) (34). After transcription, the ribozyme self-cleaves to leave a 2′−3′ cyclic phosphate end after the terminal stemloop (35). Northern blots revealed that the RiboJ constructs yielded RNA of the expected size, shifted downward from the previous constructs due to the lack of polyA tail (Fig. 5B). We then packaged these constructs into AAV2 and tested their expression *in vitro*. The 3′ end generated by RiboJ is not capable of driving translation, as the measured luciferase expression with the Luciferase-RiboJ construct was near background with only a moderate reduction in mRNA levels compared to Luciferase-polyA. However, with the addition of the DENV2 3′UTR, the reporter generated luciferase protein at roughly 60% of the level seen with polyA luciferase reporter (Fig. 5C). Ratios of RNA-normalized protein expression suggest that Luciferase-3′DENV2-RiboJ translates with roughly ~40% efficiency as the Luciferase-polyA construct, and the combined effects of stability and translation are likely additive (Fig. 5D).

### DENV2 sfRNA and DB elements increase AAV transcript stability and transgene expression *in vivo*

**Fig 6:**
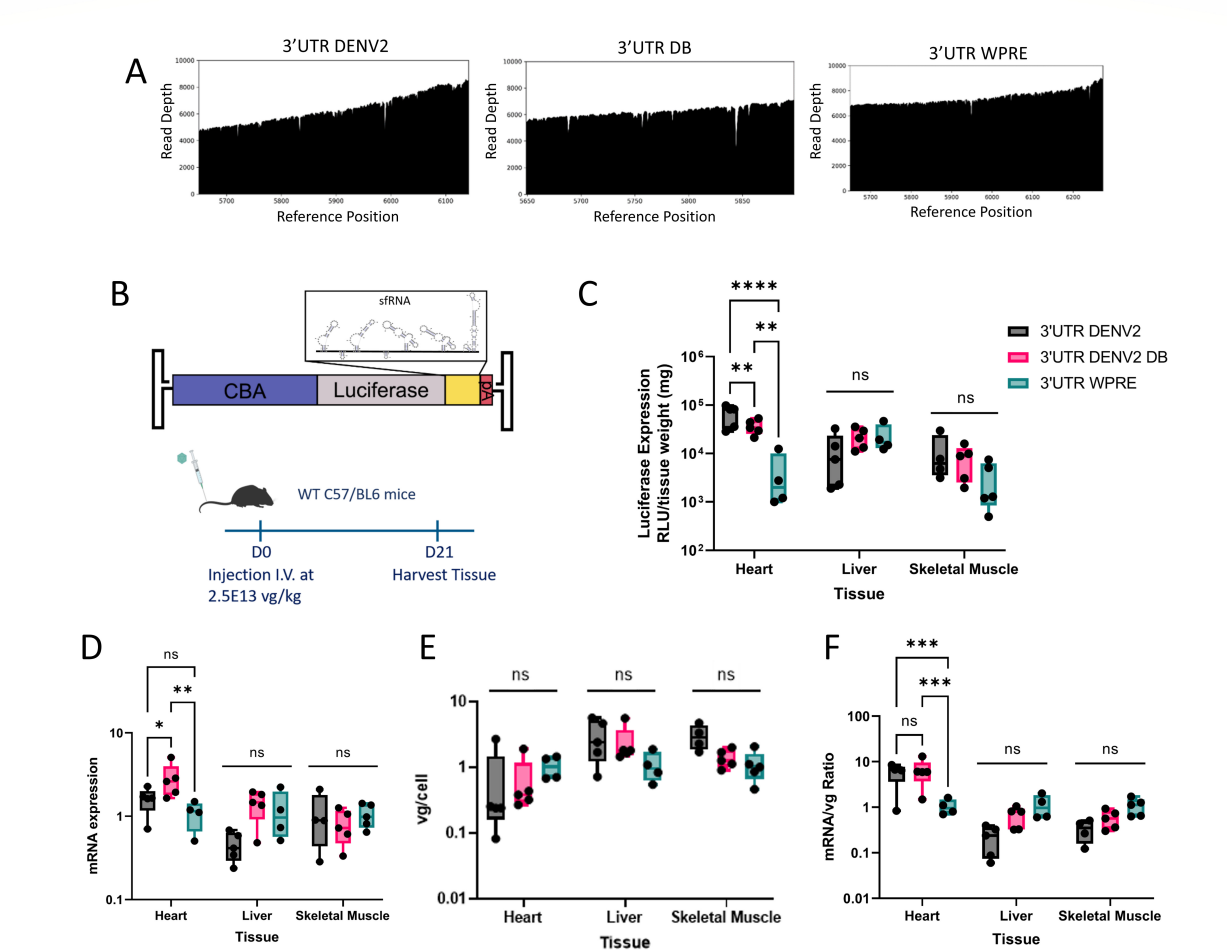
DENV2 sfRNA elements increase AAV transgene expression in mouse heart tissue *in vivo*. (**A**) Read depth at reference positions indicates vector genome coverage obtained from Nanopore analysis of AAV9 viral genomes. Plots include 3′UTR position for each of the three constructs containing DENV2, DBs, and WPRE embedded from the end of luciferase open reading frame to the beginning of SV40 polyA tail. (**B**) Eight- to ten-week old C57/BL6 female mice were injected intravenously at a dose of 2.5e13 vg/kg with AAV9. Vectors delivered consisted of a single-stranded AAV genome expressing luciferase driven by a CBA promoter and the indicated sfRNA elements (or WPRE) as the 3′UTR of the luciferase mRNA. (**C**) Tissues were harvested 3 wk post-injection, and tissue lysates were measured for relative luciferase expression (RLUs). (**D**) RT-qPCR was performed for luciferase mRNAs. Data are shown relative to mouse lamin B2 and to CBA-Luc-WPRE construct. (**E**) mRNA relative expression for each tissue piece was divided by vector genome copy number for the same tissue piece to evaluate RNA stability. (**F**) Vector biodistribution was quantitated by qPCR and calculated relative to mouse lamin B2 and to CBA-Luc-WPRE construct. For all three assays above, each dot represents an individual mouse. Box and whisker plots show median, maximum, and minimum values. Statistical significance was determined by two-way ANOVA with Holm-Sidak’s multiple comparisons test. Trends are significant where indicated, * = *P* < 0.05; ** = *P* < 0.01; *** = *P* < 0.001; **** = *P* < 0.0001.

We further evaluated the impact of DENV2 sfRNA and DB elements on AAV transcript stability and transgene expression in mice. Prior to dosing, we assessed the packaging efficiency and integrity of AAV vector genomes containing WPRE, DENV2 sfRNA, and DENV2 DB 3′UTR elements based on qPCR and Nanopore sequencing analysis. The results suggest that the AAV9 vector genome titers (ranging from 3.57 to 5.74 × 10^12^ vg/mL) and Nanopore coverage plots of these constructs are comparable to control genomes lacking 3′UTR structures (Fig. 6A). Mice (*n* = 5 for each cohort) were injected intravenously with AAV9 packaging a luciferase construct (i) tagged with a WPRE, (ii) full DENV2 sfRNA, or (iii) the two DENV2 DB structures in the 3′UTR (Fig. 6B). Heart, liver, and skeletal muscle tissues were harvested at 3 wk post-injection followed by quantitation of luciferase gene expression, relative luciferase mRNA expression, and vector genome biodistribution. Both DENV2 sfRNA and DB elements significantly increased luciferase gene expression in cardiac tissue, although this outcome was not observed in liver or skeletal muscle (Fig. 6C). Luciferase mRNA transcript levels appeared to be increased with the DB elements alone, but not the full DENV2 sfRNA construct compared to WPRE in the heart (Fig. 6D). When normalized to vector genome copies for each tissue sample, luciferase mRNA was enriched in the heart tissue for constructs with DENV2 sfRNA and DB elements in the 3′UTR compared to the control construct with WPRE (Fig. 6E). It is noteworthy to mention that no significant differences were observed in vector genome biodistribution for the various test constructs in different tissues (Fig. 6F). Thus, DENV2 sfRNA elements appear to maintain their ability to enhance transcript stability and transgene expression *in vivo*, albeit restricted to cardiac tissue in these early proof-of-concept studies.

## DISCUSSION

In this study, we examined the effect of sfRNA elements on AAV transgene expression. We found that sfRNA elements from six different flaviviruses improved transgene expression, with the DENV2 sfRNA showing the greatest effect. This effect was only observed when the DENV2 sfRNA was located in the 3′UTR of the AAV transcript, as placement in the 5′UTR or expression in *trans* showed detrimental effects on either mRNA translation efficiency or RNA abundance, respectively. We then showed that the two DBs within the sfRNA were necessary and sufficient to enhance transgene expression and that the DBs alone or the full-length sfRNA element promotes AAV transgene expression by significantly increasing mRNA stability. Overall, this study highlights the utility of viral RNA elements for stabilizing AAV transcript stability and improving transgene expression.

Flaviviral sfRNA elements accumulate throughout infection and function through multiple mechanisms to enhance virus replication (20–25). Inhibition of both XRN1 and other cellular exonucleases alters global cellular mRNA stability throughout infection (26, 27). Here, we found that the ability of sfRNA to increase mRNA levels was XRN1 independent. This was consistent with our other finding that the xrRNA elements within the sfRNA are dispensable for this function. These observations suggest that the sfRNA element is functioning through an exonuclease-independent mechanism to enhance mRNA stability.

Our results further demonstrate that the presence of only dumbbell RNA structures in the transcript 3′UTR was sufficient to increase transcript abundance. These results suggest that the DBs within the sfRNA function as an mRNA stabilizing element, possibly by interacting with RNA binding proteins (RBPs) that promote RNA stability. This could be dependent on sequence and/or RNA secondary structure within the conserved sequence 2 (CS2) region of the dumbbell structure, as mutations within the CS2 ablated the ability of the DB element to enhance AAV-mediated gene expression.

Both the full DENV2 sfRNA and DB elements alone enhanced luciferase gene expression in heart tissue compared to WPRE. As outlined earlier, it should be noted that WPRE is not expected to improve expression of high abundance transcripts driven by a CBA promoter (9). Future studies comparing the impact of sfRNA elements vs WPRE using weak/intronless promoters and cell-type specific promoters, as well as different open reading frames (ORFs) and polyA sequences, are warranted. As with WPRE, sfRNA sequences may display divergent effects when combined in different configurations for gene transfer applications. Although the effect on potency was more pronounced for the construct containing the full DENV2 sfRNA, mRNA abundance was comparable for DENV2 sfRNA and DB elements-only constructs. The boost in mRNA was also tissue specific, with full DENV2 sfRNA and DB elements alone significantly improving mRNA abundance only in cardiac tissue. Plausible explanations for such tissue-specific differences in transcript stability between different murine tissues are unclear. These may be exerted by different RBPs or host factors in transformed human cell lines, which may be different when compared to certain murine tissues. Nevertheless, these observations raise the possibility that DENV2 sfRNA and DB elements or similar structures derived from other flaviviral elements may improve transcript abundance *in vivo*. Additional evaluation in different AAV cassette configurations (e.g., self-complementary vectors) and other animal models will be required to evaluate the latter prospects.

## MATERIALS AND METHODS

### Plasmids

The primary plasmid used as a template for these studies consisted of a pUC19 cassette comprising an AAV2-based ITR-flanked luciferase reporter, which was cloned under the control of a CBA promoter and terminated by an SV40 polyA signal. WPRE or sfRNA sequences from the following viruses (DENV2 strain BR64022, GenBank AF489932.1; JEV accession number NC_001437.1; MVEV accession number NC_000943.1; WNV2 accession number NC_001563.2; YFV accession number NC_002031.1; ZIKV strain PE243, GenBank KX197192.1) were synthesized and cloned into pUC19 for propagation before cloning into the TR-CBA-Luciferase vector. This information, along with different nt lengths, is summarized in Table 1. Deletions and/or mutations in the sfRNA were constructed by site-directed mutagenesis in the pUC19 backbone before cloning into the TR-CBA-Luciferase vector. For dissecting stability and translation, the SV40 polyA signal was replaced with a synthesized RiboJ element (34, 35). The lenti-CRISPRv2 (Addgene #52961) (36) was a kind gift from Feng Zhang, and the lentiviral packaging and envelope psPAX2 (Addgene #12260) and pMD2.G (Addgene #12259) plasmids were gifts from Didier Trono.

### RNA structural depictions

RNA diagrams were drawn on RNAstructure software and rearranged on StructureEditor software (37).

### Cell culture

Cells were cultured in Dulbecco’s Modified Eagle Medium (DMEM) (Gibco) supplemented with 10% fetal bovine serum (FBS) (HEK293 and RPE cells) or 5% FBS (Huh7 and U87 cells) and 1% penicillin/streptomycin, and were maintained at 37°C and 5% CO_2_. The cells were seeded at the indicated densities in 24-well plates, and AAV was added to the media at 10,000 vg/cell unless otherwise indicated: Huh7 (1.0 × 10^4^/well), U87 (7.5 × 10^4^/well), HEK293 (4.5 × 10^4^/well), and RPE (1.0 × 10^4^/well). For larger-sized wells (12-well or 6-well), the number of cells was scaled based on area. For RiboJ transfection experiment, plasmids were added to 25 µL DMEM (Gibco). Polyethylenimine (Polysciences) was diluted in 25 µL DMEM (Gibco) in a second tube and was combined with the plasmid solution. Mixture was incubated at room temperature for 5 min and added to wells dropwise. Assays were harvested at 3 d post-transduction in either 1× Passive Lysis Buffer (Promega) for protein or TRIzol Reagent (Invitrogen) for RNA.

### Generation of CRISPR KO lines

Scramble (1:GGTCTCTGTACGGGCCGCCC and 2:TGTCATGCGTCACTTAGTGC) and XRN1 (1:TAAAACGCCTCCCACGCTGC and 2:GTATCCCTGTCTCAGCGAAG) gRNAs were cloned into the lentiCRISPRv2 backbone. Recombinant lentivirus was produced via triple plasmid transfection with psPAX2 and pMD2.G in HEK293 cells, as previously described (36). Huh7 cells were then transduced with recombinant lentivirus and subjected to puromycin selection. Extracted genomes from single clonal cell lines were PCR amplified and sequenced to verify inactivation of the XRN1 gene, and protein levels were verified by Western blot.

### Recombinant AAV vector production

Triple plasmid transfection in HEK293 cells was used to generate recombinant AAV vectors as described previously (38). Briefly, the transfection mixture contained (i) the pXR2 or pLH9 helper plasmid; (ii) the adenoviral helper plasmid pXX6-80; and (iii) the indicated transgene, driven by a CBA promoter and an SV40 polyA or a RiboJ ribozyme, flanked by AAV2 ITRs. Vector purification was carried out using iodixanol gradient ultracentrifugation followed by desalting with Zeba Spin desalting columns (40K MWCO, Thermo Scientific) or by concentration with Pierce Protein Concentrator PES columns (100K MWCO, 5–20 mL, Thermo Scientific). Detailed information on different rAAV vector genome constructs and titers of each AAV preparation is summarized in Table 1. Vector genome (vg) titers were obtained by quantitative PCR (LightCycler 480 [Roche Applied Sciences] or CFX Connect Real-Time PCR Detection System [Bio-Rad]) using SYBR Green (Roche Applied Sciences) and primers designed to selectively bind AAV2 ITRs (ITR F: AACATGCTACGCAGAGAGGGAGTGG, ITR R: CATGAGACAAGGAACCCCTAGTGATGGAG).

### Nanopore vector integrity analysis

We targeted 1E11 vg/barcode in our sequencing pipeline. The AAV vectors were first DNase treated, then the genomes were extracted by heat shocking the capsid as previously reported (38). Capsids were ruptured for genome release by the following PCR thermocycling method: first, EDTA was added to the reaction to inactivate the DNase I, DNase I was irreversibly inactivated at 65°C for 1 hr, next, the capsids were denatured by heating the samples to 95°C for 20 min, finally, the reactions were brought to a 21°C hold. The genomic material was subjected to library preparations for Oxford Nanopore Technologies (ONT) sequencing with the Native Barcoding Expansion 1-12 (EXP-NBD104) and Ligation Sequencing Kit (SQK-LSK109). Libraries were then sequenced in multiplex on an ONT MinION instrument using the R9.4.1 flow cell version. All flow cells were subjected to the recommended quality control checks before sequencing was performed. The Nanopore sequencing outputs were mapped to their reference genomes using minimap2 (v 2.24) using the map-ont preset. Aligned nucleotide read depth was performed using SAMtools (1.14-rhel8) and BEDTools (2.30.0) (39–41).

### AAV dosing *in vivo* and mouse studies

C57/BL6 mice were purchased from Jackson Laboratories (Jax#000664) and maintained at Duke University School of Medicine animal facility with the assistance of Duke’s Division of Laboratory Animal Resources (DLAR). The mice were housed in a temperature-controlled (~18–23°C, 40%–60% humidity) and enriched environment, with a 12-h light/dark cycle, and provided with standard chow and water. AAV9 was administered intravenously in female adult mice aged 8–10 wk at a dose of 2.5e13 vg/kg (*n* = 5) via the tail vein. Heart, skeletal muscle, and liver tissues were harvested 3 wk after injection for biodistribution, mRNA, and transgene expression studies.

### Luciferase assays

For *in vitro* studies, cells harvested in 1× Passive Lysis Buffer (Promega) were allowed to lyse for 30 min at room temperature. Twenty-five-microliters lysate was combined with 25-µL luciferin reagent (Promega) and measured on a Victor 3 multilabel plate reader (Perkin-Elmer). All readouts were normalized to the control CBA-Luciferase construct. For the *in vivo* study, 20–40 mg of each tissue was homogenized in 1× Passive Lysis Buffer (Promega). Forty-five-microliters luciferin (Promega) was added to 15-µL lysate, and luminescence was measured on the Victor 3 multilabel plate reader (Perkin-Elmer). Readouts were normalized to the mass of the tissue piece.

### Western blotting

Cells were harvested in 1× Passive Lysis Buffer (Promega) and stored at −20°C until use. Samples were denatured in NuPAGE LDS Sample buffer (Invitrogen) and 100 mM dithiothreitol (DTT), heated to 95°C before separation on a NuPAGE 10% Bis–Tris gel (Invitrogen), and transferred to a nitrocellulose membrane. Membranes were blocked overnight in 2% nonfat milk in Tris-buffered saline with Tween 20 (TBST). After overnight incubation, the membranes were blotted with primary antibody against either XRN1 (sc-165985, Santa Cruz Biotechnology) or Actin (1:2,000, ab3280, Abcam). Stabilized peroxidase-conjugated goat anti-mouse antibody was used as secondary antibody (1:20,000, 31430, Thermo Scientific). Blots were developed using SuperSignal West Femto Maximum Sensitivity substrate (Thermo Scientific) and exposed to film.

### Northern blotting

Ten micrograms of RNA was resuspended in denaturing buffer [67% deionized formamide, 6.7% formaldehyde, 1× 3-morpholinopropane-1-sulfonic acid (MOPS) running buffer], incubated at 65°C for 7 min, and cooled on ice. Samples were separated on a 1.2% denaturing formaldehyde-agarose gel in 1× MOPS running buffer and subsequently transferred to Hybond-N+ membrane (GE Healthcare). Radiolabeled probe was generated using the Prime-It II Random Primer Labeling Kit (Agilent Technologies) according to the manufacturer’s instructions. DNA templates for probe labeling were generated by restriction enzyme digest from plasmid template. Radiolabeled probe was purified using illustra MicroSpin G-50 columns (GE Healthcare) following the manufacturer’s protocol. The probe was then hybridized to the membrane in Rapid-Hyb buffer (GE Healthcare) at 55°C for 3 h. After washing, blots were visualized by exposure to film, and radiolabel signal was quantified by exposure to a PhosphorImager screen followed by scanning and quantification using an Amersham Typhoon (GE Healthcare). ImageQuant TL software (GE Healthcare) was used to quantify the blots using the 1D gel analysis tool; background was subtracted using the rolling circle method.

### Actinomycin treatment

HEK293 cells (2.0 × 10^5^) were seeded into 35-mm plates and transduced with 10,000 vg/cell of the indicated AAV vectors. At 3 d post-transduction, media were removed and replaced with fresh media for half an hour. These media were removed and replaced with pre-warmed and equilibrated media containing 5 µg/mL actinomycin D (Sigma-Aldrich). Cells were treated for 30 min, then media were removed and replaced with fresh media. The cells were harvested in TRIzol Reagent (Invitrogen) at 0-, 4-, 8-, 12-, and 24-h time points. RNA was extracted and analyzed by reverse transcriptase quantitative real-time PCR (RT-qPCR).

### DNA and RNA extractions

For *in vitro* studies, RNA was extracted using TRIzol Reagent (Invitrogen) following the manufacturer’s protocol; suspended in nuclease-free water and stored at −80°C until use. For *in vivo* studies, DNA and RNA were isolated from up to 60-mg tissue with Quick-DNA/RNA Miniprep Plus Kit (Zymo Research) following the manufacturer’s protocol. DNA and RNA were resuspended in nuclease-free water. DNA was stored in −20°C, and RNA was stored in −80°C until use. Two hundred nanograms of RNA was converted to cDNA with the High Capacity RNA-to-cDNA kit (Applied Biosystems).

### RNA quantitation by RT-qPCR

For *in vitro* studies, up to 5 µg of RNA was DNase treated using the Turbo DNA-free kit (Ambion). For the *in vivo* study, RNA was DNase treated on-column according to the manufacturer’s instructions. Equal nanogram amounts of DNase-treated RNA were converted to cDNA using the High Capacity RNA-to-cDNA kit (Applied Biosystems). Products of this reverse transcription reaction were utilized as template for quantitative PCR using gene-specific primers. qPCR was carried out using a Roche LightCycler 480 and SYBR Green Mastermix (Roche Applied Sciences). RT-qPCRs were quantified using the ΔΔCt method, normalized to the control CBA-Luciferase construct for *in vitro* studies or to the control CBA-Luciferase-WPRE construct for the *in vivo* study. Luciferase primers: forward (AAAAGCACTCTGATTGACAAATAC), reverse (CCTTCGCTTCAAAAAATGGAAC). Human glyceraldehyde-3-phosphate dehydrogenase (GAPDH) primers: forward (CCACTCCTCCACCTTTGAC), reverse (ACCCTGTTGCTGTAGCC). Human myc primers: forward (CGTCTCCACACATCAGCACAA), reverse (CACTGTCCAACTTGACCCTCTTG). Human TBP primers: forward (TGCACAGGAGCCAAGAGTGAA), reverse (CACATCACAGCTCCCCACCA). Mouse lamin B2 primers: forward (GGACCCAAGGACTACCTCAAGGG), reverse (AGGGCACCTCCATCTCGGAAAC).

### Quantitation of vector genomes by qPCR

Quantitative PCR was performed for *in vivo* biodistribution studies using a Roche LightCycler 480 and SYBR Green Mastermix (Roche Applied Sciences). One hundred nanograms of extracted tissue genomic DNA was added to each reaction. Results were quantified with the ΔΔCt method and normalized to the CBA-Luciferase-WPRE construct. Luciferase primers: forward (AAAAGCACTCTGATTGACAAATAC), reverse (CCTTCGCTTCAAAAAATGGAAC). Mouse lamin B2 primers: forward (GGACCCAAGGACTACCTCAAGGG), reverse (AGGGCACCTCCATCTCGGAAAC).

### Statistical analysis

For *in vitro* studies, statistical analysis was carried out using an unpaired, one-tailed Student’s *t*-test. Where indicated, n.s. = not significant, * = *P* < 0.05, ** = *P* < 0.005, *** = *P* < 0.0005, and **** = *P* < 0.00005. For in the *in vivo* study, statistical significance was determined using two-way analysis of variance (ANOVA) with Holm-Sidak multiple comparisons test. Where indicated, n.s. = not significant, * = *P* < 0.05, ** = *P* < 0.01, *** = *P* < 0.001, and **** = *P* < 0.0001. Box and whisker plots demarcate median, minimum, and maximum data points.

## Data Availability

All raw data associated with the figures in the paper are available upon request.
